# Detection of Tick-Borne Microorganisms, Anaplasmataceae and Piroplasmida, in *Sorex* spp. in Hokkaido, Japan

**DOI:** 10.3390/microorganisms13102288

**Published:** 2025-10-01

**Authors:** Aya Zamoto-Niikura, Shigeharu Terui, Mizuki Sasaki, Minoru Nakao, Masakatsu Taira, Ken-Ichi Hanaki

**Affiliations:** 1 Research Center for Biosafety, Laboratory Animal and Pathogen Bank, National Institute of Infectious Diseases (NIID), Japan Institute for Health Security (JIHS), Musashimurayama 208-0011, Tokyo, Japan; hanaki@niid.go.jp; 2 Environment Grasp Promotion Network-PEG, Nonprofit Organization, Kushiro 085-0806, Hokkaido, Japan; peg-terui@abox3.so-net.ne.jp; 3 Department of Parasitology, Asahikawa Medical University, Asahikawa 078-8510, Hokkaido, Japan; msasaki@obihiro.ac.jp (M.S.); nakao@asahikawa-med.ac.jp (M.N.); 4 Department of Veterinary Science, National Institute of Infectious Diseases (NIID), Japan Institute for Health Security (JIHS), Shinjuku 162-8640, Tokyo, Japan; taira@niid.go.jp

**Keywords:** *Babesia microti*, *Neoehrlichia mikurensis*, *Ehrlichia japonica*, *Ixodes ovatus*, *Sorex* spp., tick-borne microorganisms

## Abstract

The habitats of shrews substantially overlap with those of rodents, which are well known as reservoirs for many tick-borne diseases. However, the ecological role of shrews (Mammalia: Eulipotyphla: Soricidae) remains poorly understood. We examined 103 *Sorex* spp. (*S. unguiculatus*, *S. gracillimus*, *S. caecutiens*) from Kushiro, Hokkaido, Japan, to investigate their relationships with ticks and tick-borne microorganisms, including Piroplasmida and Anaplasmataceae. Pathogen screening revealed *Babesia microti* Hobetsu lineage (9.7%), *Neoehrlichia mikurensis* (26.2%), *Ehrlichia japonica* (13.6%), and *E. muris* (0.97%). These intracellular protozoa and bacteria, typically associated with rodents, are recognized zoonotic agents or have zoonotic potential. Detection rates were highest in *S. caecutiens* (62.5%, 10/16), followed by *S. unguiculatus* (45.3%, 24/53) and *S. gracillimus* (23.5%, 8/34). Co-infections were observed between *N. mikurensis* and *B. microti* (*n* = 3) and between *N. mikurensis* and *E. japonica* (*n* = 4). Immature stages of *Ixodes ovatus* and *I. persulcatus* were collected from the body surface of shrews, and transstadial transmission of *N. mikurensis* was suggested by its detection in a molted *I. ovatus* nymph. These results indicate that shrews act as feeding hosts for immature ticks and reservoirs for multiple tick-borne pathogens. Shrews should be considered important reservoirs for tick-borne diseases.

## 1. Introduction

Tick-borne zoonotic diseases occasionally emerge as public health threats, making it crucial to investigate potential infections in wildlife. Rodents play a crucial role as reservoir hosts for many tick-borne pathogens [1,2,3,4,5]. Recently, soricids (order Eulipotyphla), which occupy habitats similar to those of rodents, have been recognized as potentially important reservoirs for many tick-borne microorganisms, including *Babesia microti*, *Anaplasma phagocytophilum*, and various viruses [6,7,8]. Most shrews do not burrow, but inhabit areas beneath leaf litter and prey on surface-dwelling earthworms and small arthropods (insects and spiders) [9,10]. The semi-underground habitats of shrews not only overlap with the habitats of rodents but also make them a suitable host for blood meals of ticks that prefer higher humidity and lower temperatures within the leaf litter. If shrews have similar susceptibilities to infectious microorganisms to rodents, their role as reservoirs for tick-borne infectious diseases may have been overlooked [11]. Shrews are sometimes captured in traps used for small wild mammals, such as Sherman traps. However, the pitfall method, which is more labor-intensive, is required to efficiently capture shrews.

Ticks infesting shrews in Japan have been investigated in a few studies, which collected *Ixodes ovatus* and *I. persulcatus* ticks from the body surface of *Sorex* spp. [11,12]. *Ixodes ovatus* and *I. persulcatus* are the principal vectors for many tick-transmitting rodent-borne microorganisms, including *Babesia microti, Neoehrlichia mikurensis*, and *Ehrlichia japonica*.

*B. microti* is a complex of genetically diverse protozoan parasites (*B. microti*-group) of zoonotic or zoonotic potential. In the eastern part of Hokkaido, Japan (including Kushiro), previous field surveys have shown that two distinct lineages of the *B. microti* group, the Hobetsu and US lineages, were maintained by various rodent species, including *Myodes rufocanus*, and that *I. ovatus* and *I. persulcatus* ticks specifically transmitted the Hobetsu and US lineages, respectively [13,14]. The US lineage is genetically closely related to *B. microti* sensu stricto, the causative agent of human babesiosis in US. The Hobetsu lineage is widely distributed throughout Japan, and a retrospective survey suggested subclinical infection in the endemic areas of tick-borne infectious diseases [15]. In our previous study, one Hobetsu-lineage isolate was obtained from an *S. unguiculatus* sympatrically collected with the rodents in Hokkaido [16]; whether the shrew contributed to the parasite’s life cycle was unclear.

*Neoehrlichia mikurensis* was initially identified in wild rodents and ticks in Japan and is now recognized as an emerging human pathogen in Europe and Asia [3]. In Japan, the bacterium has been detected in rodents and their associated ticks (*I. ovatus* and *I. persulcatus*) with a prevalence of 9–47% [17,18]. *Ehrlichia japonica*, a newly recognized species formerly known as *Ehrlichia* sp. HF, was previously isolated from *I. ovatus* and is genetically closely related to *E. chaffeensis,* the agent of human monocytic ehrlichiosis (HME) [19]. However, no cases of infection with this organism have been reported.

The spleen is crucial for clearing blood-borne pathogens by removing damaged blood cells, capturing infected cells by macrophages, and immune activation [20]. Splenomegaly is a major clinical manifestation of *B. microti* and *Ehrlichia* infections [21,22,23]. In wild animals, the influence of these microorganism infections on the spleen has scarcely been investigated [13].

Owing to the recent emergence of infectious diseases, accumulating data on tick vectors and their reservoir hosts is critical for public health. In this study, we actively investigated the potential role of shrews as hosts for ticks and reservoirs for tick-borne obligate intracellular pathogens, including *Babesia, Neoehrlichia,* and *Ehrlichia.* Additionally, we investigated splenomegaly in relation to infection with these microorganisms.

## 2. Materials and Methods

### 2.1. Collection of Wild Shrew

**Figure 1:**
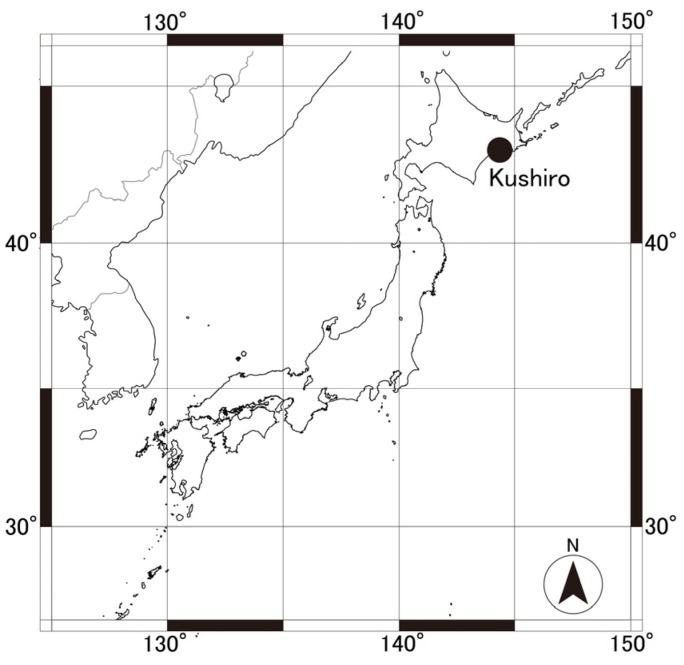
Map of the survey area (Kushiro, Hokkaido, Japan).

Shrews were unintentionally collected during salamander surveys in Kushiro Wetland in Hokkaido, Japan, in October 2023 (Figure 1) and dead bodies were provided. In the survey, a set of 75 pitfall traps with drift fences were placed. Species were primarily identified based on their morphological characteristics [24]. The fifth unicuspid tooth (U5) of the upper jaw was examined to discriminate between *S. caecutiens* and *S. gracillimus* [25], as the appearance of immature *S. caecutiens* was similar to that of adult *S. gracillimus* [24]. Additionally, genetic examination was performed based on the *cyt-b* sequence (780 bp) [26] using DNA from the spleen (see below). Spleens were dissected using disposable tweezers and blades and stored in alcohol until use. To eliminate cross-contamination, the dissection instruments were changed for each individual. An apparently enlarged spleen (e.g., a spleen covering the entire abdominal cavity) was regarded as splenomegaly.

### 2.2. Collection of Ticks

Ticks were collected from the body surface of *Sorex* spp. before dissection. Additionally, ticks that remained on the shrew’s body were collected from the dissected body. The body was tightly enclosed in a plastic bag for a few hours at room temperature. The ticks that crawled into the plastic bag were collected. All collected ticks were stored in moistened plastic tubes at room temperature. Engorged ticks were kept alive in tightly sealed plastic tubes over a moistened filling of solidified plaster with activated charcoal at 25 °C until molting. Ticks were collected in 2020 at the same location in October using the same method described above. The ticks collected in 2020 were kept in alcohol until species identification.

### 2.3. DNA Extraction from Ticks and Spleen of Shrews

The spleens of wild shrews were cut into approximately 100 mg pieces and homogenized in tubes containing a ceramic ball (Ceramic Sphere, MP Biomedicals, Solon, OH, USA; catalog no. 6540412), garnet beads (Garnet Matrix A Bulk, MP Biomedicals; catalog no. 6540-427), and TNE buffer [50 mM Tris–HCl (pH 7.4), 100 mM NaCl, and 0.1 mM EDTA] by shaking at 4000 rpm for 30 s using a Micro Smash MS-100 (TOMY, Katsushika, Tokyo, Japan). Proteinase K (0.1 mg/mL) and sodium dodecyl sulfate (0.1%) were then added, and the samples were incubated overnight at 55 °C. DNA was extracted using the standard phenol/ethanol method, dissolved in 200 μL of TE buffer [10 mM Tris–HCl (pH 7.4) and 1 mM EDTA], and stored at −30 °C.

Ticks were homogenized with plastic pestles in TNE buffer containing Proteinase K (0.1 mg/mL) and sodium dodecyl sulfate (0.1%). After incubation at 55 °C for three hours, DNA was extracted from the ticks using a standard protocol based on the phenol/ethanol method [27].

### 2.4. Microorganism Detection in Ticks and Spleens

Ex Taq polymerase (Takara Bio, Otsu, Japan) was used according to the manufacturer’s instructions.

Piroplasmids were detected using nested polymerase chain reaction (PCR) targeting the *18S rRNA* gene, as described by Tsuji et al. [13]. The 18S rRNA-positive samples were further examined for the presence of *Babesia microti* and lineage identification. Since three *B. microti* lineages are distributed in wild rodents in Japan, the lineage was discriminated using discriminative PCR based on the *β-tubulin* sequence [28].

Members of the Anaplasmataceae family were identified by dual gene sequencing based on the *16S rRNA* and *groEL* genes [29]. Nested PCR and direct sequencing were performed using the primers listed in Table S1. BLAST (v.2.16.0) was used to search for high sequence similarity to known species. A positive result for Anaplasmataceae identification was confirmed by the high sequence similarity of both the *16S rRNA* and *groEL* genes to those of a known species in the same specimen. In cases where different species (e.g., *Neoehrlichia* and *Ehrlichia*) were identified based on *16S rRNA* and *groEL* sequences from the same specimen, further analyses were conducted. PCRs targeting *Ehrlichia P28* [17] and *Neoehrlichia mikurensis*-specific *16S rRNA* genes [18] were performed in a separate laboratory. Co-infection was confirmed when multiple pathogens were identified in both laboratories. All primers used in this study are listed in the Supplementary Table S1.

### 2.5. Phylogenetic Analysis

For phylogenetic analysis, the sequences determined in this study and those retrieved from GenBank were aligned using ClustalW. Evolutionary relationships were inferred using the neighbor-joining method implemented in MEGA X software (version 11.0.13) [30]. Bootstrap values were estimated for 1000 replicates and are indicated at the branch. An outgroup was chosen from species which are closely related to the group of interest.

### 2.6. Statistical Test

The chi-squared test was used to examine statistically significant differences between the observed and expected frequencies of splenomegaly. The test was performed for each microorganism-positive group individually, for the combined Anaplasmataceae-positive group (*N. mikurensis*, *E. japonica*, and *E. muris*), and for all microorganism-positive shrews (*B. microti*, *N. mikurensis*, *E. japonica*, and *E. muris*).

## 3. Results

### 3.1. Collection and Phylogeny of Sorex spp.

**Figure 2:**
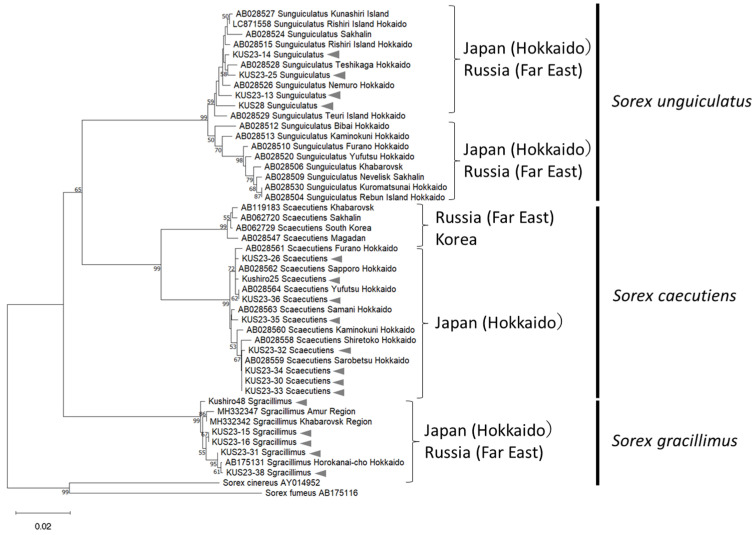
Phylogenetic tree based on the mitochondrial *cyt-b* gene sequence (780 bp, NJ method). Bootstrap values (%) of 1000 times are shown at branching points (>50%). The representative sequences from *Sorex* spp. collected in Kushiro are indicated by arrows. The sequences from GenBank are shown with accession numbers.

In total, 103 *Sorex* spp. specimens were collected, including *S. unguiculatus* (*n* = 53), *S. gracillimus* (*n* = 34), and *S. caecutiens* (*n* = 16). Two shrew specimens that were morphologically identified as *S. caecutiens* were molecularly identified as *S. gracillimus*, while one shrew specimen morphologically identified as *S. gracillimus* was molecularly identified as *S. caecutiens*, indicating that molecular identification is crucial for accurately differentiating these species. A phylogenetic tree of *Sorex* spp. based on the *cytb* showed that *S. gracillimus* and *S. caecutiens* collected in Kushiro in this study formed a distinct lineage with those previously reported individuals collected in Hokkaido, Japan, and were clearly distinguished from those found in Russia (Figure 2). In contrast, the *S. unguiculatus* consisted of two lineages, regardless of the collection area, including Hokkaido and Russia, consistent with previous findings by Odachi et al. [31]. Since *S. unguiculatus* is thought to have distributed across northeastern Eurasia more recently than *S. gracillimus* and *S. caecutiens*, insufficient nucleotide substitutions may have accumulated to reflect geographical isolation [31].

### 3.2. Immature Ixodes ovatus and I. persulcatus Feed on Sorex spp.

Sixty-four ticks were collected from 12 shrews (5.3 ticks/shrew) (Table 1). The collection included the immature stages of two ixodid ticks, *I. ovatus* (*n* = 28) and *I. persulcatus* (*n* = 36), but not the adult stage. Both tick species were collected from all three species of *Sorex*. *I. persulcatus* (56%) and *I. ovatus* (44%) were collected in relatively equal numbers. The mean numbers of *I. ovatus* and *I. persulcatus* collected from shrews were 2.3 ticks/shrew and 3 ticks/shrew, respectively. Owing to the small sample size, statistical analysis to assess the correlations between *Sorex* host species and bloodsucking tick species was not performed. As in the 2020 collection, all ticks (*n* = 148) were immature stages of *I. ovatus* (*n* = 101) and *I. persulcatus* (*n* = 47) (Table 1). The mean number of ticks collected from the shrews was 9.9 ticks/shrew.

### 3.3. Molecular Detection of Piroplasmida and Anaplasmataceae in Ticks

Ticks collected from the body surface of *Sorex* spp. were kept in containers for molting. After 1 month or more, a total of 21 nymphs molted from the larvae (Table 2). From four *S. unguiculatus* and two *S. caecutiens,* 15 and 6 nymphs were obtained, respectively. The tick species and numbers collected from each shrew are listed in Table 2.

PCR examination for microorganisms in the ticks revealed a Piroplasmida-positive *I. persulcatus* (designated as KUS23-33_m3) and an Anaplasmataceae-positive *I. ovatus* (designated as KUS23-33_m5). Both ticks were recovered from same *S. unguiculatus* (designated KUS23-33) in which *N. mikurensis* was detected (see below). Further PCR for lineage discrimination of *B. microti* in the piroplasmida-positive specimen (KUS23-33_m3) failed to identify the species, as amplification was not observed with any primer sets (Table S1). PCR amplicon of 18S rRNA obtained during the initial screening was subjected to sequencing. In the BLAST search, the resulting *18S rRNA* (1519 bp) of KUS23-33_m3 was most closely related to that of the *B. divergens* Asia lineage (KC493555.1), with 99.87% identity. The *I. persulcatus* tick KUS23-33_m3 infected with the *B. divergens* Asia lineage most likely originated from an *I. persulcatus* female infected with this lineage, since *B. divergens* is both transstadially and transovarially transmitted through a multi-stage life cycle, and this lineage is primarily maintained by wild deer and *I. persulcatus* in Hokkaido [27].

**Figure 3:**
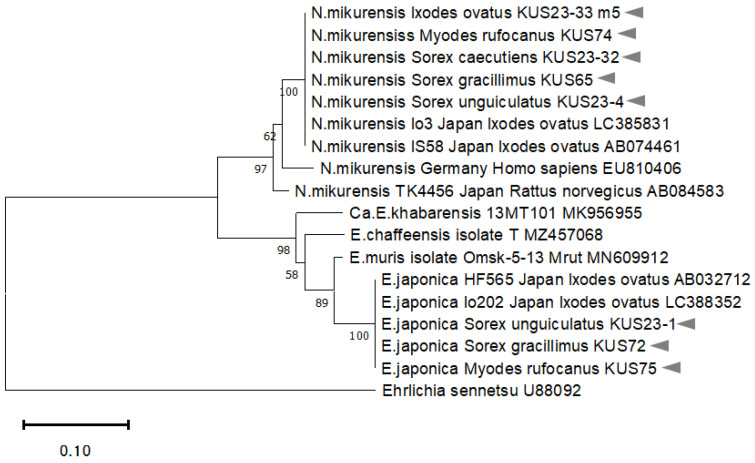
Phylogenetic tree based on the *groEL* sequence (282 bp) using the NJ method. Bootstrap values (%) of 1000 times are shown at branching points. The sequences determined in this study are indicated by the arrows.

*GroEL* sequence (290 bp) and *16S rRNA* (756 bp) of the Anaplasmataceae-positive specimen (KUS23-33_m5) was identical to that of *N. mikurensis* Io3 from *I. ovatus* in Japan (LC385831) and *N. mikurensis* FIN686 (AB196304), respectively (Figure 3).

### 3.4. Detection of Babesia microti, Neoehrlichia mikurensis, Ehrlichia japonica, and E. muris in Sorex spp. Spleen

DNA extracted from the spleen was examined for the presence of tick-borne organisms, including the Piroplasmida and Anaplasmataceae species. The positivity rates for each microorganism in *Sorex* spp. are shown in Table 3. Piroplasmida *18S rRNA*-positive samples were classified as belonging to the *B. microti* Hobetsu lineage using lineage-specific PCR. Sequencing of all Anaplasmataceae *GroEL*-positive samples (*n* = 38) revealed three distinct sequences (Table 4). One was identical to the *groEL* of *N. mikurensis* Io3 from *I. ovatus* in Japan (LC385831) (*n* = 23), and the others were identical to that of *Ehrlichia* sp. HF565 (*E. japonica*) isolated from *I. ovatus* (AB032712) (*n* = 14) and *E. muris* (*n* = 1). The *N. mikurensis* sequence was also identical to those of the IS58 strain from wild rats (AB074461) and the A113 strain from wild rodents (AB204865) in Japan (Figure 3). An identical sequence of *N. mikurensis* was also detected in sympatrically captured *Myodes rufocanus* (KUS75) (Figure 3). The *16S rRNA* sequences were identified as those of *N. mikurensis* (*n* = 27), *E. japonica* (*n* = 10), or *E. muris* (*n* = 1), which corresponded to the results obtained from *groEL*, except for four samples (*S. unguiculatus*, *n* = 3; *S. gracillimus*, *n* = 1).

In the four spleen samples, *groEL* and *16SrRNA* sequences were of different microorganisms: *groEL* sequences were identified as *E. japonica*, whereas *16S rRNA* sequences were identified as *N. mikurensis*. Co-infection with these organisms was further confirmed in a separate laboratory using PCR assays targeting *N. mikurensis 16S rRNA* and *Ehrlichia P28*. The nearly full-length *16S rRNA* sequences from all four samples were identical to each other and to that of *N. mikurensis* strain IS58 (AB074460). All four *P28* sequences were identified as *E. japonica*, although the chromatograms of the central region were ambiguous. Co-infection with *N. mikurensis* and *B. microti* was observed in *S. caecutiens* (*n* = 2) and *S. unguiculatus* (*n* = 1). Co-infection data are summarized in Table 4.

### 3.5. Detection of Splenomegaly in Shrews

**Figure 4:**
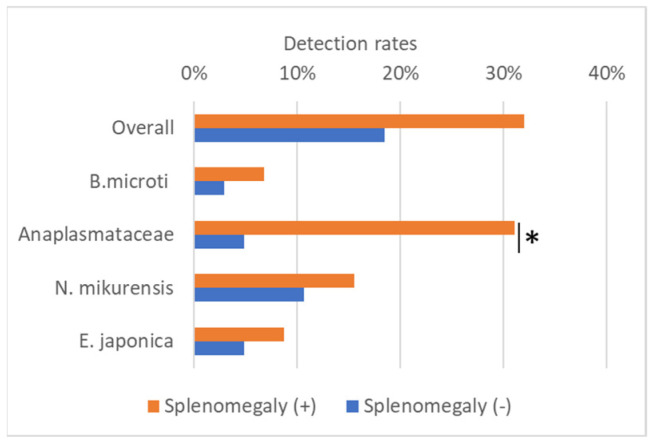
Detection of Piroplasmida and Anaplasmataceae in splenomegaly-positive (*n* = 66) and -negative (*n* = 37) *Sorex* spp. The Anaplasmataceae family includes *Neoehrlichia* and *Ehrlichia.* Significant differences between splenomegaly-positive and -negative groups are represented by an asterisk (tested by Chi-Square Test, * *p* < 0.05).

Splenomegaly was observed in 66 *Sorex* spp. examined (64.1%). Positive rates in each *Sorex* spp. were 90.6% (48/53) in *S. unguiculatus,* 35.3% (12/34) in *S. gracillimus*, and 37.5% (6/16) in *S. caecutiens*. Splenomegaly was highly frequent in *S. unguiculatus*. Necropsy revealed no visible nodules in the spleen. The number of splenomegaly-positive shrews was higher than that of splenomegaly-negative shrews for all infections (Figure 4). In particular, the number of splenomegaly-positive shrews was statistically higher in the Anaplasmataceae (*N. mikurensis, E. japonica*, and *E. muris*)-positive shrews (Chi-Square Test, *p* < 0.05).

## 4. Discussion

In this study, we investigated the presence of *Sorex* spp. in Hokkaido to explore their potential role as reservoirs of tick-borne microorganisms and the tick species involved in their transmission. Molecular screening targeting Piroplasmida and Anaplasmataceae in *Sorex* spp. spleen identified *B. microti* Hobetsu lineage, *E. japonica*, *N. mikurensis*, and *E. muris* (Table 3). Co-infection of *N. mikurensis* and *B. microti,* the most frequent co-infection case in wild rodents [28], was observed in *Sorex* spp. (Table 3). Ticks collected from the body of the shrew (Table 1) were of *Ixodes ovatus* and *I. persulcatus,* which are the principal vectors of *B. microti* Hobetsu lineage (*I. ovatus*) [14], *N. mikurensis* (*I. ovatus* and *I. persulcatus*) [17,19], *E. japonica* (*I. ovatus*) [32], and *E. muris* (*I. persulcatus*) [33]. These results indicate that both shrews and rodents serve as the reservoirs for tick-borne microorganisms, including *B. microti* Hobetsu lineage, *E. japonica*, *N. mikurensis*, and *E. muris,* and that *I. ovatus* and *I. persulcatus* ticks transmit the transmission of these microorganisms.

The results of this study showed that the infection rate of the Hobetsu lineage in shrews (9.7%, 10/103; Table 3) was comparable to that observed in rodents [16]. Furthermore, this lineage was detected in all three predominant species of *Sorex* spp. in Hokkaido (Table 4). Thus, we conclude that *Sorex* spp. play a major role in maintaining *B. microti* Hobetsu lineage reservoirs. We speculate that the extensive distribution of the Hobetsu lineage throughout Japan, from north (Hokkaido) to south (Shikoku), is due to divergent mammalian reservoirs, including *Sorex* spp., and the geographically wide distribution of the competent vector (*I. ovatus*). In the USA [34,35] and UK [6], *Sorex* spp. are regarded as competent US lineage (or *B. microti* sensu stricto) reservoirs. The reason why the US lineage has not yet been detected in *Sorex* spp. in this country is unclear. Although, *I. persulcatus*, the principal US-lineage vector, was collected from *Sorex* spp. as efficiently as *I. ovatus* (Table 2).

We found that *Sorex* spp. also harbored *N. mikurensis*, with particularly high prevalence in *S. caecutiens* (43.8%) and *S. unguiculatus* (30.2%; Table 3), comparable to rodent infection rates. Regarding the previous detection of *N. mikurensis* in *S. unguiculatus* (2/4) [36], our findings suggest that shrews are important reservoirs for the natural maintenance of *N. mikurensis* in Hokkaido. Notably, *N. mikurensis* undergoes vertical (transplacental) transmission in rodents, supporting its persistence in small mammalian populations, even in the absence of tick feeding [36]. Although vertical transmission of *N. mikurensis* has been confirmed in rodents, similar mechanisms in shrews remain speculative and require further investigation. Combined with the tick preference for shrews (Table 2) and confirmed transstadial transmission (Table 3), *I. ovatus* and *I. persulcatus* likely play pivotal roles in maintaining and bridging *N. mikurensis* among both the shrew and rodent populations in Hokkaido.

In this study, *E. japonica* was detected in *S. unguiculatus* and *S. gracillimus*, with the highest prevalence in *S. unguiculatus* (20.8%) (Table 3). Notably, co-infection with *N. mikurensis* was confirmed in several individuals using both *groEL* and *P28* analyses. This co-infection may be due to sharing the same vector tick, *I. ovatus* [18,37], which feeds *on Sorex* spp. (Table 1). The strong association between *E. japonica* infection and splenomegaly (Figure 4) is consistent with the immunostimulatory effects observed in canine ehrlichiosis caused by *E. canis* [22,38], suggesting its potential pathogenicity. Considering the wide geographical distribution of *I. ovatus*, continued surveillance of *E. japonica* in wildlife reservoirs and tick populations is necessary.

We speculated that the observed splenomegaly was associated with infections by *B. microti*, *Ehrlichia* spp., and/or *N. mikurensis* [39], given that splenomegaly was present in as many as 64.1% of the examined shrews. However, our data did not provide statistical support for an association between *B. microti* and the detection of other microorganisms (Figure 4). The co-detection of *Babesia*, *Ehrlichia*, and *Neoehrlichia* in our samples suggests that a dynamic community of tick-borne pathogens is maintained within the shrew population. Therefore, the splenomegaly observed in this study may not be attributable to a single pathogen, but rather may reflect chronic immune stimulation driven by the diverse assemblage of tick-borne pathogens present in the spleen.

The collection of immature *I. ovatus* and *I. persulcatus* from shrews (Table 1) corresponds to a report in which *S. unguiculatus* and sympatrically captured *A. speciosus* were investigated in October in Furano (approximately 150 km away from Kushiro) [11]. October may restrict tick collection in terms of stage, as adult ticks that overwinter are active in the spring. Only immature *I. ovatus* and *I. persulcatus* were primarily shown to feed on rodents, *Apodemus* spp. and *Myodes* spp., through the tick-active period (5 months) in Kiyosato and Shimizucho (approximately 100 km away from Kushiro) [40]. Ongoing global warming, ecology, and the distribution of ticks may change, making it necessary to conduct continuous surveys for a comprehensive understanding of wild shrews as feeding hosts.

Shrews have been increasingly identified as an important reservoir of zoonotic viral infections [6,7]. The results of this study emphasize the involvement of *Sorex* spp. as the reservoir of protozoa and bacteria, including *B. microti* Hobetsu lineage [14], *E. japonica* [19,32], and *N. mikurensis* [32,37]. Although clinical cases of human babesiosis caused by *B. microti* Hobetsu lineage have not been documented, the presence of antibodies against this lineage in a high-risk group [15] suggests subclinical infection. Ongoing, geographically expanded surveys encompassing a wide range of pathogens are essential for clarifying the ecological role of shrews as reservoirs and preparing for emerging infectious disease outbreaks.

## 5. Conclusions

The examination of ticks attached to shrews and the detection of *Babesia microti* Hobetsu lineage, *Neoehrlichia mikurensis*, and *Ehrlichia japonica* in *Sorex* spp. in Kushiro, Hokkaido, Japan, indicate that shrews serve as feeding hosts for immature *I. ovatus* and *I. persulcatus* ticks and as predominant reservoirs for these tick-borne microorganisms. These findings indicate that *Sorex* spp. may play a pivotal role in the transmission dynamics of tick-borne pathogens. To clarify the ecological significance of shrews as potential risk factors for tick-borne diseases, continuous and geographically expanded surveillance, encompassing a broader spectrum of pathogens, will be required.

## Figures and Tables

**Table 1:** Tick collection from *Sorex* spp.

	*Sorex* spp.	*Ixodes ovatus*		*I. persulcautus*			
		Larva	Nymph	Larva	Nymph	Total	Tick/Shrew
2023	*S. unguiculatus* (*n* = 10)	11	8	11	18	48	4.8
	*S. gracillimus* (*n* = 1)	0	1	0	0	1	1
	*S. caecutiens* (*n* = 1)	3	5	3	4	15	15
2020	*S. unguiculatus* (*n* = 10)	83	8	41	0	132	13.2
	*S. gracillimus* (*n* = 5)	8	2	6	0	16	3.2

**Table 2:** Detection of Piroplasmida and Anaplasmataceae in molted ticks.

*Sorex* spp. ^1^	Resulted Nymph			
	*I. ovatus*		*I. persulcatus*	
	No. Examined	Microorganism Detected	No. Examined	Microorganism Detected
*S. unguiculatus*	1	- ^2^	0	-
*S. unguiculatus*	7	-	5	*Babesia divergens* Asia lineage
*S. unguiculatus*	1	-	0	-
*S. unguiculatus*	0	-	1	-
*S. caecutiens*	2	*Neoehrlichia mikurensis*	3	-
*S. caecutiens*	1	-	0	-

^1^ Ticks were collected from each shrew (total of 6). ^2^ Not detected.

**Table 3:** Detection rate of Piroplasmida and Anaplasmataceae in the spleen of *Sorex* spp.

*Sorex* spp.	No. Examined	*B. microti*		*N. mikurensis*		*E. japonica*		*E. muris*	
*S. unguiculatus*	53	3 ^1^	(5.7%)	16 ^1,2^	(30.2%)	11 ^2^	(20.8%)	0	(0%)
*S. gracillimus*	34	3	(8.8%)	4 ^3^	(11.8%)	3 ^3^	(8.8%)	1	(2.9%)
*S. caecutiens*	16	4 ^4^	(25.0%)	7 ^4^	(43.8%)	0	(0%)	0	(0%)

^1^ Containing mixed infection of *B. microti* and *N. mikurensis* (*n* = 1). ^2^ Containing mixed infections of *N. mikurensis* and *E. japonica* (*n* = 3). ^3^ Containing mixed infection of *N. mikurensis* and *E. japonica* (*n* = 1). ^4^ Containing mixed infection of *B. microti* and *N. mikurensis* (*n* = 2).

**Table 4:** Detection of multiple tick-borne microorganisms in *Sorex* spp.

Microorganisms Co-Infected	*S. unguiculatus*	*S. gracillimus*	*S. caecutiens*	Total
*Neoehrlichia mikurensis* and *Ehrlichia japonica*	3	1	0	4
*N. mikurensis* and *Babesia microti*	1	0	2	3

## Data Availability

Representative sequences obtained in this study were deposited in the DNA Data Bank of Japan (DDBJ) under accession numbers LC877955–LC877961 *(N. mikurensis*, *16S rRNA*), LC867973–LC867977 (*N. mikurensis*, *GroEL*), LC877954 *(E. japonica*, *16S rRNA*), LC867978–LC867980 (*E. japonica*, *GroEL*), and LC871563–LC871578 (*Sorex* spp., *Cyt*-b).

## Supplementary Materials

The following supporting information can be downloaded at https://www.mdpi.com/article/10.3390/microorganisms13102288/s1, Table S1: Primers used for PCR in this study.

## Abbreviation

The following abbreviation is used in this manuscript:

PCR	Polymerase chain reaction
