# Supplementary material for: Detection of Tick-Borne Microorganisms, Anaplasmataceae and Piroplasmida, in Sorex spp. in Hokkaido, Japan
Source: Microorganisms. 2025 Oct 1;13(10):2288. doi: 10.3390/microorganisms13102288 (PMC12566115; doi:10.3390/microorganisms13102288)
Supplement: Supplementary file 1 [file microorganisms-13-02288-s001.zip › microorganisms-3806847-supplementary.pdf]

Table S1. Primers used for PCR in this study.

Genes	Primers	1st/2nd	Sequences (5' - 3')	References
Sorex spp., cytochrome-b,	Rodent Cyt-b_F1 (ap)	1st	GACYAATGACATGAAAAATCATCGTTG	Zamoto-Niikura et al. 2023
	RodentCyt-b_R1 (ap)	1st	AGAATATCAGCTTTGGGTGCTGATGGTGGGG	
	RodentCyt-b_F2 (ap, s)	2nd	ATCATCGTTGTWATTCAACTATAGAAAC	
	RodentCyt-b_R2 (ap, s)	2nd	CATTACMYTGGTCTTGTAAACCARAAATG	
Anaplasmataceae 16S rRNA	E16S-5F (ap)	1st	AGAACGAACGCTRGCGGYAAGC	Tabara et al. 2007
	E16S-3R (ap)	1st	ACCCYAGTCACYVACCCMACC	
	E16S-200F (ap, s)	2nd	GATCAGCCACACTGGAACTGAGA	
	E16S-1162R (ap, s)	2nd	CATTGTAGCACGTGTGTAGCCCA	
Anaplasmataceae groEL	gro607F (ap)	1st	GAAGATGCWGTWGGWTGTACKGC	Tabara et al. 2007
	gro1249R (ap)	1st	AGMGCTTCWCCTTCWACRTCYTC	
	gro667F (ap, s)	2nd	ATTACTCAGAGTGCTTCTCARTG	
	gro1121R (ap, s)	2nd	TGCATACCRTCAGTYTTTTCAAC	
Ehrlichia P28	conP28-F1 (ap)	1st	ATYAGTGSAAARTAYRTRCCAA	Inayoshi et al. 2004
	conP28-R1 (ap)	1st	TTARAARGYAAAYCTKCCTCC	
	conP28-F2 (ap, s)	2nd	CAATGGRWGGYCCMAGARTAG	
	conP28-R2 (ap, s)	2nd	TTCCYTGRTARGMAAKTTTAGG	
N. mikurensis 16S rRNA	Eh-out1 (ap)	1st	TTGAGAGTTTGATCCTGGCTCAGAACG	Li et al. 2012.
	3–17U (ap)	1st	WAAGGWGGTAATCCAGC	
	EHR16SD (ap, s)	2nd	GGTACCYACAGAAGAAGTCC	
	EHR16SR (ap, s)	2nd	TAGCACTCATCGTTTACAGC	
	Eh-out2U (ap, s)	2nd	CACCTCTACACTAGGAATTCCACTATC	
	Eh-out2fU (ap, s)	2nd	GATAGTGGAATTCCTAGTGTAGAGGTG	
	CNM1050f (s)		TAACCCTTGTCCTTAGTTGCC	
Piroplasminda 18SrRNA	Piro 0F (ap)	1st	GCCAGTAGTCATATGCTTGTGTTA	Tsuji et al. 2006
	Piro6R (ap)	1st	CTCCTTCCTTAAGTGATAAGGTTCAC	
	Piro 1F (ap, s)	2nd	CCATGCATGTCTWAGTAYAARCTTTTA	
	Piro5.5R (ap, s)	2nd	CCTYTAAGTGATAAGGTTCACAAAACTT	
B.microti beta-tubulin	Tubu-63F (ap)	1st	CAAATWGGYGCMAARTTYTGGGA	Tsuji et al. 2006
	Tubu-3R (ap)	1st	TCGTCCATACCTTCWCCSGTRTACCAGTG	
	Tubu-ATG5F (ap)	2nd	ATGAGAGARATYGTACACATYCAAGC	
	Tubu-1538R (ap)	2nd	TAYTGYTGGTAYTCGCTRACYA	
	Tubu-Ho5' (ap)	2nd	AAGAGCTAACGTTTTTTACAATCTATCAAG	Zamoto et al. 2004
	Tubu-Ho3' (ap)	2nd	CGCAAATCCAATCATAAAAAAGTTTAGTC	
	Tubu-Ko5' (ap)	2nd	CAAATGTTTTTTATAACCAGACGAGCG	
	Tubu-Ko3' (ap)	2nd	GAAAGGAATAAGATTCACAGTGAGCT	
	Tubu-US5' (ap)	2nd	GCAAAYGTTTTYTATAACCAGTTTAGTG	
	Tubu-US3' (ap)	2nd	GAAATGCAATCTCGGGAAGGTAATGA	

ap, amplification primer; s, sequencing primer
